# Bioaccessibility of Carotenoids and Polyphenols in Organic Butternut Squash (*Cucurbita moschata*): Impact of Industrial Freezing Process

**DOI:** 10.3390/foods13020239

**Published:** 2024-01-11

**Authors:** Senem Kamiloglu, Elif Koc Alibasoglu, Busra Acoglu Celik, M. Alpgiray Celik, Erturk Bekar, Taha Turgut Unal, Buket Kertis, Arzu Akpinar Bayizit, Perihan Yolci Omeroglu, O. Utku Copur

**Affiliations:** 1 Department of Food Engineering, Faculty of Agriculture, Bursa Uludag University, Bursa 16059, Türkiye; elifkoc0894@gmail.com (E.K.A.); busraacoglu@gmail.com (B.A.C.); erturk@uludag.edu.tr (E.B.); tahaunal@uludag.edu.tr (T.T.U.); buketkertis98@gmail.com (B.K.); abayizit@uludag.edu.tr (A.A.B.); pyomeroglu@uludag.edu.tr (P.Y.O.); ucopur@uludag.edu.tr (O.U.C.); 2 Science and Technology Application and Research Center (BITUAM), Bursa Uludag University, Bursa 16059, Türkiye; alpgiray@uludag.edu.tr; 3 Department of Food Hygiene and Technology, Faculty of Veterinary Medicine, Bursa Uludag University, Bursa 16059, Türkiye

**Keywords:** *Cucurbita moschata* var. Butternut, individual quick freezing, industrial waste, in vitro digestion, *β*-carotene, epicatechin, syringic acid, UPLC-ESI-MS/MS, HPLC-PDA

## Abstract

Butternut squash (*Cucurbita moschata*) is recognized as a functional food due to its abundant content of health-promoting compounds, including carotenoids and polyphenols. The aim of this study was to examine the impact of industrial freezing stages on the bioaccessibility of carotenoids and polyphenols in organic Butternut squash supplied for baby food. Identification and quantification of bioactive compounds were carried out using UPLC-ESI-MS/MS and HPLC-PDA, respectively. The results revealed that industrial freezing of squash did not cause a significant change in bioaccessibility of *α*- and *β*-carotene. On the other hand, frozen squash was found to contain higher levels of bioaccessible epicatechin (main flavonoid) (117.5 mg/kg) and syringic acid (main phenolic acid) (32.0 mg/kg) compared to fresh internal fruit. Moreover, the levels of bioaccessible epicatechin and syringic acid were found to be the highest in discarded pomace and seed sample (454.0 and 132.4 mg/kg, respectively). Overall, this study emphasized that industrial freezing could be an effective strategy for preserving carotenoid bioaccessibility in organic Butternut squash, while also enhancing the levels of bioaccessible polyphenols. In addition, we also demonstrated that pomace and seed, which are discarded as waste, have significant potential to be utilized as a food source rich in bioactive compounds.

## 1. Introduction

*Cucurbita* species are recognized for their significant health-promoting activities attributed to the bioactive properties of the compounds they contain. The most well-known biological activities of the *Cucurbita* species include antioxidant [1], antimicrobial [2], antidiabetic [3], and anticarcinogenic [4] effects. Butternut squash (*Cucurbita moschata*), a subspecies of the *Cucurbita* genus, is acknowledged as a functional food due to its abundant content of health-promoting compounds, including vitamins, minerals, and phytochemicals [5]. Among the phytochemical compounds in Butternut squash, carotenoids and polyphenols are acknowledged as the primary contributors that have a beneficial effects on health.

According to the most recent data of the Food and Agriculture Organization (FAO), global squash production, including pumpkins and gourds, has reached approximately 24 million tons. Türkiye holds the sixth position among the world’s top-producing countries with an annual production of 771,651 tons [6]. The produced squash, especially the Butternut variety obtained through organic farming, has various industrial applications including commercial baby food production. The lasting impact of nutrition during the initial thousand days of life is widely acknowledged. The phase of complementary feeding represents a crucial period where environmental factors, including feeding practices, can influence infant development, exerting long-term effects that may extend into adulthood. In industrialized countries, the rise in employed mothers and evolving lifestyles has led to the development of various commercial baby foods [7]. The nutritional quality of commercial baby foods is of utmost importance for infant’s health.

Butternut squash used as an ingredient in commercial baby food is commonly supplied from industrial food processors after having undergone peeling, dicing, and freezing processes. Individual quick freezing (IQF) is widely employed in the industry as a technique that rapidly freezes products, reducing ice crystal size and effectively preserving the nutritional content of fruits and vegetables [8]. During the IQF process of Butternut squash, various waste products are generated, including peels, pomace, and seeds. The disposal of such waste products in the industry not only has the potential to create adverse environmental impacts but also poses a significant cost for the industry. On the other hand, multiple studies have indicated that some industrial food wastes can be utilized as sources of bioactive compounds including carotenoids and polyphenols [9,10,11]. Moreover, pumpkin seed, belonging to the same family as squash, may be consumed fresh or roasted as appetizers, and is quite popular in Türkiye with the production of 42.000 tons per year [12,13]. One of the 17 sustainable development goals set by the United Nations to be achieved by 2030 involves promoting waste reduction among industries, businesses, and consumers [14]. Therefore, exploring the potential utilization of industrial waste products is of great importance.

The beneficial effects of carotenoids and polyphenols on health greatly rely on their bioaccessibility within the gastrointestinal tract. In vitro gastrointestinal digestion models have been developed to explore the bioaccessibility of carotenoids and polyphenols from various food sources. These models offer the benefits of being quick, cost-effective, and free from ethical constraints encountered in human studies [15]. Despite the presence of a limited number of papers examining the changes in the bioaccessibility of bioactive compounds as a result of industrial freezing process [16,17,18], to the best of our knowledge no previous study investigated the changes in the bioaccessibility of carotenoids and polyphenols in organic Butternut squash at different stages of the industrial freezing process. Similarly, although there are several studies in the literature that have applied in vitro gastrointestinal digestion models directly to fruit and vegetable wastes [19,20,21,22], this study stands out as the first to investigate the bioaccessibility of carotenoids and polyphenols from squash wastes.

Given the above considerations, this study aimed to examine the impact of industrial freezing stages on the bioaccessibility of carotenoids and polyphenols in organic Butternut squash supplied for baby food. Identification and quantification of bioactive compounds were achieved using UPLC-ESI-MS/MS and HPLC-PDA, respectively. Furthermore, spectrophotometric assays for total phenolic and flavonoid content, along with total antioxidant capacity, were also employed to assess changes during the simulation of in vitro gastrointestinal digestion.

## 2. Materials and Methods

### 2.1. Materials

**Figure 1:**
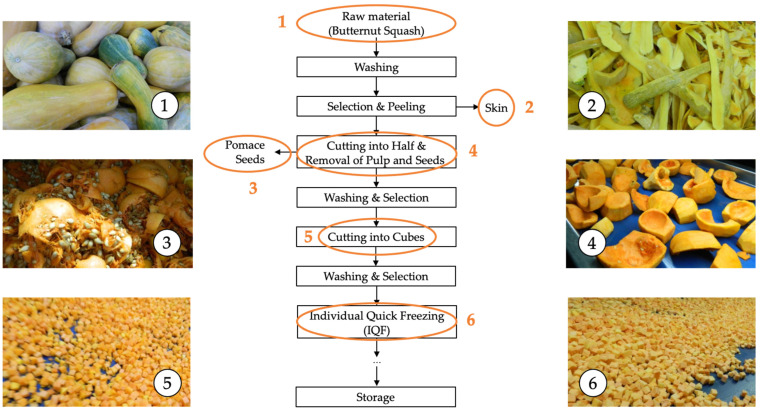
The flowchart summarizing the stages involved in the industrial freezing process of organic butternut squash (*Cucurbita moschata*). 1: Raw material, 2: Skin, 3: Pomace and seeds, 4: Raw material without skin and seeds (internal fruit), 5: Cutting, 6: Individual quick freezing (IQF).

The organic Butternut squash (*Cucurbita moschata*), harvested at a commercially mature stage in the Polatli district of Ankara, was transported to the food processing facility (Mevsim Gida Co., Salihli, Manisa, Türkiye), where they were subjected to an industrial freezing process using the individual quick freezing (IQF) (Frigoscandia FloFreeze^®^ MX, Helsingborg, Sweden) technique according to the flow chart provided in Figure 1. The cutting procedure employed prior to freezing process refers to obtaining 10 × 10 mm pieces using an industrial food cutter (Urschel DiversaCut Sprint^®^ Dicer, Chesterton, IN, USA). The final product is supplied as raw material to a renowned baby food manufacturer. Within the scope of this study, samples collected on three different production days from six points indicated in the flowchart were transferred to the laboratory with a cold chain to minimize quality loss. The collected samples were ground into a fine powder using a grinder (Büchi Mixer B-400, Flawil, Switzerland) and stored in a −80 °C freezer (Ultra-Low Temperature Freezer MDF-U5386S, Panasonic Co., Osaka, Japan) until analysis.

### 2.2. Chemicals

For the simulation of in vitro gastrointestinal digestion potassium chloride, sodium bicarbonate (Isolab, Istanbul, Türkiye), monopotassium phosphate (Honeywell, Charlotte, NC, USA), magnesium chloride hexahydrate, hydrochloric acid, sodium hydroxide (Merck, Darmstadt, Germany), calcium chloride (Tekkim, Bursa, Türkiye), sodium chloride, ammonium carbonate, *α*-amylase, pepsin, pancreatin, and bile (Sigma-Aldrich, Steinheim, Germany) were used.

For the extraction of carotenoids and polyphenols, analytical grade hexane, acetone, ethanol, tetrahydrofuran, methanol and formic acid (Merck) were used, and for their chromatographic analysis, liquid chromatography grade methanol, acetonitrile (Merck) and trifluoroacetic acid (Sigma-Aldrich) were used. The following analytical standards were used for the quantification of carotenoids and polyphenols: *α*-carotene (≥97%), *β*-carotene (≥95%), gallic acid (≥98.0%) (Sigma-Aldrich), chlorogenic acid, syringic acid, ferulic acid, epigallocatechin, epicatechin, luteolin 7-*O*-glucoside, quercetin 3-*O*-galactoside, quercetin 3-*O*-glucuronide (TRC, Ontario, Canada), and naringin (90.5%) (Dr. Ehrenstorfer, Augsburg, Germany).

For the determination of total phenolic and flavonoid contents and total antioxidant capacity, Folin–Ciocalteu reagent, sodium carbonate, sodium nitrite, aluminum chloride (Merck), 2,2–azinobis(3–ethylbenzothiazoline–6–sulfonic acid diammonium salt) (ABTS), neocuproine, 2,4,6–tripyridyl–s–triazine (TPTZ), iron (III) chloride, 2,2-diphenyl-1-picrylhydrazyl (DPPH) (Sigma-Aldrich), rutin, 6-hydroxy-2,5,7,8-tetramethylchroman-2-carboxylic acid (Trolox^®^) (Acros Organics, Fair Lawn, NJ, USA), monopotassium phosphate, dipotassium phosphate (Carlo Erba, Milan, Italy) and ammonium acetate (Isolab) were used.

### 2.3. In Vitro Gastrointestinal Digestion

A three-stage in vitro gastrointestinal digestion model, simulating oral, gastric, and intestinal digestion, was applied to determine the bioaccessibility of carotenoids and polyphenols in organic Butternut squash. The oral, gastric, and intestinal electrolyte solutions were prepared as specified in the protocol [23,24].

To simulate oral digestion, 10.00 ± 0.01 g of the sample was mixed with 7 mL of oral electrolyte solution, 1 mL of *α*-amylase (1500 U/mL), 0.05 mL of 0.3 M calcium chloride, and 1.95 mL of distilled water. The mixture was placed in a shaking water bath (Nüve ST 30, Ankara, Türkiye) and incubated at 37 °C for 2 min. After completing oral digestion, 4 mL of aliquot was taken for each sample.

To simulate gastric digestion, 12 mL of gastric electrolyte solution, 2.56 mL of pepsin (25,000 U/mL), and 0.008 mL of 0.3 M calcium chloride were added to the remaining solution. The pH was adjusted to 3 using 1 M of hydrochloric acid. Then, the total volume was completed to 16 mL with the addition of distilled water, and the mixture was incubated in a shaking water bath at 37 °C for 2 h. After completing gastric digestion, 4 mL of aliquot was taken for each sample.

To simulate intestinal digestion, the remaining mixture was combined with 15.4 mL of intestinal electrolyte solution, 7 mL of pancreatin (800 U/mL), 3.5 mL of 160 mM bile, and 0.056 mL of 0.3 M calcium chloride. The pH was adjusted to 7 using 1 M sodium hydroxide. Then, distilled water was added to achieve a total volume of 28 mL, and the mixture was incubated in a shaking water bath at 37 °C for an additional 2 h. After completing intestinal digestion, 4 mL of aliquot was again taken for each sample.

In addition to the above, incubations were performed under the same conditions without adding organic Butternut squash samples, and the collected blank aliquots were used to correct for interactions arising from digestion fluids.

### 2.4. Carotenoids

#### 2.4.1. Extraction

The extraction procedure of carotenoids was carried out by modifying the method previously described in the literature [25]. An amount of 2.00 ± 0.01 g of the undigested and digested sample was mixed with 2 mL hexane:acetone:ethanol (50:25:25, *v*/*v*/*v*) and shaken roughly. After shaking, the mixture was centrifuged at 10,000× *g* for 10 min at 4 °C (Hitachi CF15RN, Tokyo, Japan). The upper phase was transferred to a clean tube, evaporated under nitrogen, and stored at −20 °C until analysis. Prior to chromatographic analysis, the residue remaining at the bottom of the tube was dissolved in tetrahydrofuran:methanol (50:50, *v*/*v*).

#### 2.4.2. Identification and Quantification Using High Performance Liquid Chromatography-Photodiode Array Detector (HPLC-PDA)

For the identification and quantification of carotenoids, all samples were filtered through 0.45 μm membrane filters and injected into the HPLC-PDA (Shimadzu LC-2030 C, Tokyo, Japan) equipped with a C18 column (25 cm × 4.6 mm, 5 μm; Macherey-Nagel, Düren, Germany) as previously described in the literature [25]. The flow rate for spectral measurement at 475 nm with a PDA detector was set at 1 mL/min, and the injection volume was 10 μL. Methanol:acetonitrile (90:10, *v*/*v*) was used as the mobile phase. The column temperature was maintained at 30 °C, and the program duration was 35 min. Retention times and characteristic spectra were considered for the identification of carotenoids. The carotenoids were quantified using external standards. The calibration curves showed good linearity (*R*^2^ > 0.999) within the determined range (1–200 mg/L). Limit of detection (LOD) and limit of quantification (LOQ) were 0.3 and 1.0 mg/L, respectively. The results were expressed as mg/kg.

### 2.5. Polyphenols

#### 2.5.1. Extraction

The extraction procedure of polyphenols was conducted as previously described in the literature [16]. A total of 2.00 ± 0.01 g of undigested sample was mixed with 5 mL of a 75% methanol solution containing 0.1% formic acid and kept in a cooled ultrasonic bath for 15 min (Elma LC30H, Darmstadt, Germany). Subsequently, the mixture was centrifuged at 10,000× *g* for 10 min at 4 °C. The upper phase was transferred to a clean tube, and the same procedure was repeated once more. The two upper phases were combined, and the final volume was adjusted to 10 mL. Digested samples were stabilized by adjusting the pH to 2 with formic acid and centrifuged at 20,000× *g* and 4 °C for 10 min, and the supernatants were injected into UPLC-ESI-MS/MS and HPLC-PDA for identification and quantification, respectively. The prepared extracts were stored in a −20 °C freezer until analysis.

#### 2.5.2. Identification Using Ultra High Performance Liquid Chromatography-Electrospray Ionization-Tandem Mass Spectrometry (UPLC-ESI-MS/MS)

For the identification of polyphenols, all samples were filtered through 0.22 μm membrane filters and injected into the UPLC-ESI-MS/MS (Shimadzu LC–MS/MS 8060, Kyoto, Japan) equipped with a C18 column (10 cm × 3 mm, 3 μm; GL Sciences, Tokyo, Japan) as previously described in the literature [26]. The flow rate was set at 0.4 mL/min and the injection volume was 10 μL. MQ water with formic acid (1000:1, *v*/*v*) and acetonitrile with formic acid (1000:1, *v*/*v*) were used as mobile phase A and B, respectively. The gradient was as the following: 0 min, 20% B; 0.0–0.5 min, 20% B, isocratic; 0.5–7.0 min, 20–50% B, linear; 7.0–12.0 min, 50–95% B, linear; 12.0–12.1 min, 95–20% B, linear; 12.1–15.0 min, 20% B, isocratic. The column and autosampler temperatures were maintained at 40 °C and 10 °C, respectively. The instrument parameters were the following: nebulizing gas (N_2_) flow at 3.0 L/min, drying gas (N_2_) flow at 10.0 L/min, interface voltage set to 4.0 kV, desolvation line temperature maintained at 250 °C, interface temperature set at 300 °C, and the heat block temperature at 400 °C. The MS/MS system was operated in both negative and positive ion modes with multiple reaction monitoring (MRM) using the ESI source. 

#### 2.5.3. Quantification Using HPLC-PDA

For the quantification of polyphenols, all samples were filtered through 0.45 μm membrane filters and injected into the HPLC-PDA equipped with a C18 column (25 cm × 4.6 mm, 5 μm; MZ-Analysentechnik, Mainz, Germany) as previously described in the literature [16]. The flow rate for spectral measurements at 280, 312 and 360 nm with a PDA detector was set at 1 mL/min, and the injection volume was 10 μL. MQ water with trifluoroacetic acid (1000:1, *v*/*v*) and acetonitrile with trifluoroacetic acid (1000:1, *v*/*v*) were used as mobile phase A and B, respectively. The gradient was the following: 0 min, 5% B; 0–45 min, 35% B; 45–47 min, 75% B; 47–49 min, 35% B; 50 min, 5% B. The column and autosampler temperatures were maintained at 40 °C and 10 °C, respectively. Retention times and characteristic spectra were considered for the identification of polyphenols. The polyphenols were quantified using external standards. Table S1 in Supplementary Materials shows the analytical parameters of the polyphenol standards within the determined range (LOQ-100 mg/L). The results were expressed as mg/kg.

### 2.6. Total Phenolic Content

The total phenolic content was determined as previously described in the literature [27]. After adding 0.75 mL of Folin–Ciocalteu reagent to 100 μL of the extract, the mixture was incubated at room temperature for 5 min. Following incubation, 0.75 mL of 6% sodium carbonate solution was added, and the mixture was kept at room temperature for 90 min. Subsequently, absorbance at 725 nm was measured using the UV-Vis spectrophotometer (Agilent Cary 60, Santa Clara, CA, USA). The total phenolic content of the samples was determined using a gallic acid standard curve (linear range: 10–600 mg/L, *R^2^* = 0.999), and the results were expressed as mg gallic acid equivalent (GAE)/100 g of sample.

### 2.7. Total Flavonoid Content

The total flavonoid content was determined as previously described in the literature [28]. After adding 0.3 mL of 5% sodium nitrite solution to 1 mL of the extract, the mixture was incubated at room temperature for 5 min. Following incubation, 0.3 mL of 10% aluminum chloride solution was added, and 1 min later, 2 mL of 1 M sodium hydroxide was added to the mixture. Subsequently, 2.4 mL of distilled water was added, and absorbance was measured at 510 nm using the UV-Vis spectrophotometer. The total flavonoid content of the samples was determined using a rutin standard curve (linear range: 1–800 mg/L, *R^2^* = 0.997), and the results were expressed as mg rutin equivalent (RE)/100 g sample.

### 2.8. Total Antioxidant Capacity

#### 2.8.1. ABTS Assay

The ABTS assay was applied as previously described in the literature [29]. The ABTS stock solution was diluted to an absorbance of 0.90 ± 0.05 at 734 nm in 50 mM pH 8 potassium phosphate buffer. Subsequently, 100 μL of the extract was mixed with 1 mL of the ABTS working solution, and after 1 min of incubation, absorbance was measured at 734 nm using the UV-Vis spectrophotometer. The total antioxidant capacity of the samples was determined using a Trolox^®^ standard curve (linear range: 1–60 mg/L, *R^2^* = 0.998), and the results were expressed as mg Trolox^®^ equivalent (TE)/100 g sample.

#### 2.8.2. Cupric Ion Reducing Antioxidant Capacity (CUPRAC) Assay

The CUPRAC assay was applied as previously described in the literature [30]. A total of 100 μL of the extract was sequentially mixed with 1 mL of 10 mM copper (II) chloride, 1 mL of 7.5 mM neocuproine, 1 mL of 1 M ammonium acetate, and 1 mL of distilled water to reach a final volume of 4.1 mL. After incubation at room temperature for 30 min, absorbance was measured at 450 nm using the UV-Vis spectrophotometer. The total antioxidant capacity of the samples was determined using a Trolox^®^ standard curve (linear range: 10–800 mg/L, *R^2^* = 0.999), and the results were expressed as mg Trolox^®^ equivalent (TE)/100 g sample.

#### 2.8.3. Ferric Ion Reducing Antioxidant Power (FRAP) Assay

The FRAP assay was applied as previously described in the literature [31]. A total of 100 μL of the extract was mixed with 900 μL of FRAP reagent (a mixture of pH 3.6 acetate buffer, 10 mM TPTZ solution, and 20 mM iron (III) chloride in a ratio of 10:1:1, *v*/*v*/*v*). After 4 min of incubation at room temperature, absorbance was measured at 593 nm using the UV-Vis spectrophotometer. The total antioxidant capacity of the samples was determined using a Trolox^®^ standard curve (linear range: 1–200 mg/L, *R^2^* = 0.999), and the results were expressed as mg Trolox^®^ equivalent (TE)/100 g sample.

#### 2.8.4. DPPH Assay

The DPPH assay was applied as previously described in the literature [32]. A total of 100 μL of the extract was mixed with 2 mL of 0.1 mM DPPH reagent dissolved in methanol. After 30 min of incubation at room temperature, absorbance was measured at 517 nm using the UV-Vis spectrophotometer. The total antioxidant capacity of the samples was determined using a Trolox^®^ standard curve (linear range: 10–100 mg/L, *R^2^* = 0.996), and the results were expressed as mg Trolox^®^ equivalent (TE)/100 g sample.

### 2.9. Statistical Analysis

All analyses were conducted by performing three measurements on each of the samples obtained in triplicate, and the results were expressed as mean ± standard deviation. The data were subjected to one-way analysis of variance (ANOVA) using SPSS 24.0 software (IBM, Chicago, IL, USA), and Tukey’s test was used to determine differences between the samples (*p* < 0.05).

## 3. Results and Discussion

### 3.1. Carotenoids

**Figure 2:**
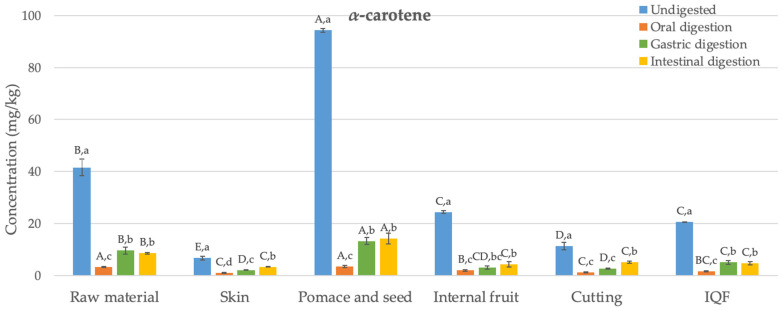

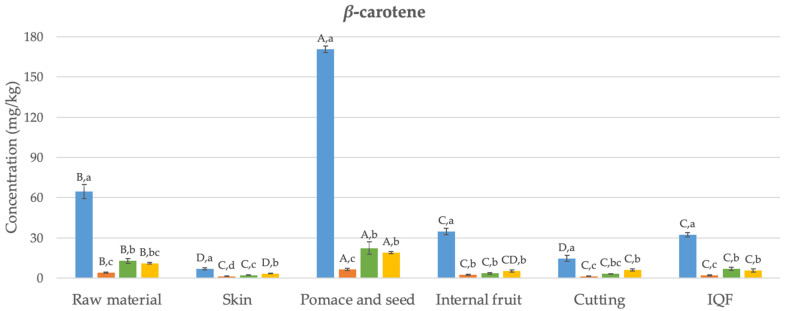
Changes in the bioaccessibility of carotenoids in organic Butternut squash (*Cucurbita moschata*) at six different stages of industrial freezing process. The data presented in these figures consist of average values ± standard deviation of three independent batches (n = 3). Different capital and small letters above bars represent statistically significant differences between samples and digestion stage, respectively (*p* < 0.05).

The main carotenoids detected in samples collected from six different stages during the industrial freezing process of organic Butternut squash (*Cucurbita moschata*) were *α*- and *β*-carotene (Figure 2). This finding is consistent with some previous studies on Butternut squash in the literature [33]; however, in other studies, in addition to *α*- and *β*-carotene, lutein and violaxanthin have also been detected [34]. The variation observed among these studies may be attributed to differences in factors, such as fruit ripeness, applied agricultural practices, storage conditions including temperature, duration from harvesting to processing, and the presence of factors like oxygen or light.

The levels of *α*- and *β*-carotene in the undigested raw material were determined as 42 and 65 mg/kg, respectively. Studies in the literature have reported similar results to these values (24–67 mg/kg) [35]. Generally, the concentration of *β*-carotene was higher than that of *α*-carotene, as found in this study. The carotenoid content in the skin was significantly lower compared to the internal fruit (by 73% and 80% for *α*- and *β*-carotene, respectively) (*p* < 0.05). In their study, Hussain et al. [36] also found that the skin of Butternut squash had a lower level of *β*-carotene compared to the internal fruit. Pomace and seed, which are discarded as waste, have been found as the richest samples in terms of carotenoids, with *α*- and *β*-carotene contents of 94 and 171 mg/kg, respectively. Although the cutting process has reduced the *α*- and *β*-carotene content (by 54% and 58%, respectively) (*p* < 0.05), statistically, no significant difference was observed in terms of carotenoid content between the undigested final IQF product and the internal fruit (*p* > 0.05). Similarly, studies conducted with carrots [37] and apricots [38] also reported that the freezing process preserves the content of *α*- and *β*-carotene.

Figure 2 illustrates the changes in the bioaccessibility of carotenoids in organic Butternut squash at six stages of the industrial freezing process. Generally, concentrations of *α*- and *β*-carotene determined after oral digestion were statistically significantly lower than those found in undigested samples (84–96%) (*p* < 0.05). After gastric digestion, despite detecting 1–16 mg/kg higher amounts of *α*- and *β*-carotene compared to the oral digestion (*p* < 0.05), the levels of *α*- and *β*-carotene remained statistically significantly lower than those in undigested samples (68–90%) (*p* < 0.05). Following the completion of intestinal digestion, the levels of *α*- and *β*-carotene were generally not statistically different from the data observed after gastric digestion (*p* > 0.05). The bioaccessibility values obtained after completing digestion ranged between 11 and 50%. The highest bioaccessibility values were determined in the waste product consisting of pomace and seed for both *α*- and *β*-carotene (14 and 19 mg/kg, respectively). The obtained bioaccessible carotenoid levels were statistically significantly higher compared to the raw material (64–72%) (*p* < 0.05). On the other hand, no difference was observed in terms of carotenoid bioaccessibility between the internal fruit, samples obtained after cutting, and the final IQF product (*p* > 0.05). This indicates that the industrial freezing process did not cause a statistically significant change in carotenoid bioaccessibility.

As indicated above, the levels of released carotenoids after oral digestion were quite low. Similar findings were reported in a carotenoid bioaccessibility study conducted with mandarin [39]. After gastric digestion, low levels of carotenoids were determined again compared to undigested samples. A previous study examining the bioaccessibility of carotenoids in mango suggested that after gastric digestion, fruit cells and vascular fibers remained intact, continuing to encapsulate carotenoids. This means that acid hydrolysis may not play a significant role in breaking down the cell wall and releasing carotenoids [40]. The bioaccessibility values obtained for the raw material after intestinal digestion (17–21%) are consistent with the bioaccessibility values of *α*- and *β*-carotene previously reported in a study conducted with Butternut squash (16–18%) [34]. Additionally, the bioaccessibility values determined for *α*- and *β*-carotene in other fruits and vegetables, such as carrot (20–22%), mango (32–33%), papaya (49%), sweet potato (14%), tomato (16%), and watermelon (30%) [34,40], also fall within a similar range to the bioaccessibility values found in the current study (11–50%).

The low bioaccessibility of carotenoids during digestion may result from the oxidation of these compounds [41]. Indeed, under in vivo conditions, carotenoids can undergo transformations as they pass through the gastrointestinal system. For instance, *β*-carotene can be cleaved in the intestinal mucosa to produce vitamin A. In this study, no new metabolites were identified after completing gastrointestinal digestion. Previous studies have suggested that in vitro digestion conditions might generate low molecular weight colorless degradation compounds [39]. It is possible that these compounds were undetectable at the wavelength measured in this study (475 nm).

The bioaccessibility of carotenoids is influenced by several factors, one of which is the variety of the product subjected to digestion. Studies on mango [42] and mandarin [39] concluded that the bioaccessibility of *β*-carotene varies within the ranges of 16–36% and 25–39%, respectively, for different varieties or genotypes. Another factor affecting carotenoid bioaccessibility is particle size. For instance, when mango is consumed in puree form, the bioaccessibility of *β*-carotene has been observed to increase from 33% to 67% [40]. Likewise, in case of carrots, the bioaccessibility of *β*-carotene was 3%, while for carrot juice, it was determined to be 14% [43]. The food matrix is yet another factor influencing the bioaccessibility of carotenoids. It is suggested that dietary fibers present in the food matrix can disrupt the formation of micelles during intestinal digestion by separating bile salts. In the study by Aschoff et al. [44], the bioaccessibility of carotenoids found in fresh orange slices (11%) was lower than the value observed for fruit juice (29–30%), and this difference was attributed to the high fiber content of the fruit. Similarly, in our study, the bioaccessibility of carotenoids determined for pomace and seed (11–15%) was lower than the values determined for other samples (15–50%).

Food processing is a major factor that influences carotenoid bioaccessibility. There are several studies in the literature suggesting that heat treatment leads to increased micellization, thereby enhancing the bioaccessibility of carotenoids. For instance, the bioaccessible *β*-carotene detected in cooked carrots (52%) was significantly higher than the value found for raw carrots (29%) [45]. Similarly, the bioaccessibility of *β*-carotene in certain red pepper varieties was determined to be higher after boiling compared to the raw material [46]. On the other hand, the bioaccessibility of carotenoids in frozen peppers was found to be similar to that in unprocessed peppers [47]. Confirming this finding, in this study we also observed that the industrial freezing process did not cause a significant change in carotenoid bioaccessibility. 

### 3.2. Polyphenols

The UPLC–ESI–MS/MS analysis of organic Butternut squash samples led to the identification of 10 major polyphenols including six flavonoids and four phenolic acids (Table 1). Most of the polyphenols were detected in negative mode, with the exception of epigallocatechin, which is identified in positive mode. The identification process involved consideration of MS, fragmentation patterns, and comparison with existing literature [48,49,50,51,52,53,54].

#### 3.2.1. Flavonoids

In undigested samples, the major compounds, representing 86–99% of total flavonoids, were identified as epicatechin and epigallocatechin (Table 2). Consistent with this finding, Mansour et al. [53] also identified epicatechin and epigallocatechin as the predominant polyphenols in pumpkin byproducts. In contrast to carotenoids, the undigested skin exhibited a significantly higher flavonoid content than the internal fruit (25%) (*p* < 0.05). Notably, the pomace and seed demonstrated the highest flavonoid content among the analyzed samples (465.7 mg/kg) (*p* < 0.05). The cutting process led to a reduction in flavonoid levels (53%), and furthermore, the undigested final IQF product contained lower levels of flavonoids compared to the internal fruit (49%) (*p* < 0.05). In fact, some of the minor flavonoids, including luteolin 7-*O*-glucoside, quercetin 3-*O*-galactoside and quercetin 3-*O*-glucuronide, vanished after the cutting step of the industrial freezing process. The loss of these glycosylated flavonoids may also be attributed to their cleavage to aglycones [55]. The detrimental effect of industrial freezing on glycosylated flavonoids has been documented in previous studies, particularly in oranges, wherein narirutin and rutin levels exhibited a significant reduction after industrial freezing (53–65%) (*p* < 0.05) [18].

The alterations in flavonoid bioaccessibility in organic Butternut squash throughout various stages of the industrial freezing process are outlined in Table 2. The levels of flavonoids determined following oral digestion were significantly lower compared to those observed in undigested samples (75–89%) (*p* < 0.05). Luteolin 7-*O*-glucoside, quercetin 3-*O*-galactoside and naringin were absent in all samples at all stages of in vitro gastrointestinal digestion. Similarly, epigallocatechin was undetectable for digested internal fruit, cut and IQF samples, which could be related to its cleavage to epicatechin. In fact, the levels of epicatechin increased during gastric and intestinal digestion, ultimately reaching significantly higher levels than those in undigested samples for the majority of the samples (up to 68%). Likewise, quercetin 3-*O*-glucuronide levels were also significantly higher in digested raw material, skin and pomace and seed, as compared to their undigested counterparts (35–75%). The enhanced level of some flavonoids following intestinal digestion may also be attributed to the prolonged contact time of food material with intestinal fluids, facilitating the release of these flavonoids by intestinal enzymes.

The bioaccessibility of epicatechin and quercetin 3-*O*-glucuronide were found to be the highest in the discarded pomace and seed sample (168% and 175%, respectively). Moreover, the IQF sample contained higher bioaccessible epicatechin compared to internal fruit (157% and 71%, respectively). The development of ice crystals during the freezing process causes damage to the food structure, potentially leading to the breakdown of cell walls. These modifications in the matrix may enhance the release of flavonoids during in vitro gastrointestinal digestion. The positive impact of the industrial freezing process on the bioaccessibility of flavonoids also has been observed in previous studies involving strawberry [16] and lemon peel [9].

#### 3.2.2. Phenolic Acids

The major phenolic acids present in undigested samples were identified as syringic and gallic acids, accounting for 70–94% of total phenolic acids (Table 3). Similarly, Stryjecka et al. [54] also detected syringic acid as the main phenolic acid in five different pumpkin species including Butternut squash. Confirming the findings obtained for flavonoids, undigested skin contained higher levels of phenolic acids compared to internal fruit (23%) (*p* < 0.05) and pomace and seed had the highest concentration of phenolic acids (180.1 mg/kg) (*p* < 0.05). Although the total phenolic acid content decreased after the cutting treatment (33%) (*p* < 0.05), there was statistically no significant difference in the phenolic acid content of IQF and cut samples (*p* > 0.05). The reduction in phenolic compounds following cutting process may be related to the fact that cutting could activate biochemical defense mechanisms, causing phenolic substrates in the vacuole to interact with enzymes, resulting in browning. Furthermore, this process may accelerate ripening biochemistry, leading to the softening of the texture [56]. Nevertheless, Yang et al. [51] also could not detect syringic acid in pumpkins subjected to the cooking process.

Table 3 presents the changes in the bioaccessibility of phenolic acids in organic Butternut squash throughout six different stages of the industrial freezing process. Similar to the results obtained for flavonoids, the levels of phenolic acids determined following oral digestion were significantly lower compared to those observed in undigested samples (79–93%) (*p* < 0.05). Minor phenolic acids, i.e., ferulic and chlorogenic acids, were either undetected or found in low amounts during the entire in vitro gastrointestinal digestion process. Gastric digestion increased syringic acid levels compared to oral digestion, with further release observed during intestinal digestion. As indicated above for flavonoids, the enhanced level of syringic acid following digestion may also be related to the increased contact time of squash with intestinal fluids, enabling the release of bioactive compounds by intestinal enzymes. Among the analyzed samples, the pomace and seed sample exhibited the highest levels of bioaccessible syringic acid (210%). Similarly, the only sample demonstrating the presence of bioaccessible gallic and chlorogenic acids was pomace and seed (12% and 135%, respectively). Furthermore, similar to the result obtained for flavonoid epicatechin, the IQF sample contained higher bioaccessible syringic acid compared to internal fruit (160% and 81%, respectively).

### 3.3. Total Phenolics, Flavonoids and Antioxidant Capacity

The total phenolic contents determined for the undigested raw material and peel (Table 4) were higher than the values previously reported in the literature [57,58]. As indicted above for carotenoids, these differences may be related to variations in factors, such as fruit ripeness, applied agricultural practices, storage conditions including temperature, duration from harvesting to processing, and the presence of factors like oxygen or light. The peel exhibited a higher total flavonoid content compared to the internal fruit. Similarly, in their study with pumpkins, Singh et al. [59] also observed that the total flavonoid content in the fruit peel was higher compared to the internal fruit. Among the analyzed samples, pomace and seed contained the highest levels of total phenolics, flavonoids and antioxidant capacity, which is consistent with the findings reported in previous studies [50,51]. The cutting process reduced the total phenolics, flavonoids and antioxidant capacity (4–80%), and frozen squash was found to contain lower levels of total phenolics, flavonoids and antioxidant capacity compared to internal fruit (7–46%). The reduction in phenolics and other antioxidants observed after cutting can be attributed to oxidative stress on the fruit, resulting in membrane damage. Furthermore, the formation of ice crystals during the freezing process may lead to a loss of turgor pressure in cells and damage to the cell wall structure. The decline in total phenolics and antioxidants observed during the freezing step may be linked to this phenomenon [60].

The changes in the bioaccessibility of total phenolics, flavonoids and antioxidant capacity in organic Butternut squash throughout different stages of the industrial freezing process are presented in Table 4. As in chromatographic polyphenol analysis, the levels of total phenolics, flavonoids and antioxidant capacity after oral digestion were generally significantly lower compared to those observed in undigested samples (*p* < 0.05). The limited release of phenolics, flavonoids and other antioxidants during oral digestion may be attributed to the brief contact time (2 min) with the enzyme [61]. In fact, following gastric and intestinal digestion levels of total phenolics, flavonoids and antioxidant capacity increased, reaching to even higher levels than of undigested samples. These increases may be attributed to the prolonged extraction time (2 h each) and/or the impact of intestinal digestive enzymes on the complex food matrix, promoting the release of phenolics bound within the matrix [62]. Moreover, breakdown of phenolic compounds may form new metabolites with higher antioxidant potential [63]. The shift from low to neutral pH during gastrointestinal digestion may also contribute to enhanced antioxidant activity [64]. There was statistically no significant difference between frozen squash and internal fruit in terms of total phenolic and flavonoid contents and total antioxidant capacity (*p* > 0.05).

In this study, four different antioxidant capacity measurement assays (ABTS, CUPRAC, DPPH, and FRAP) were applied to determine the changes in the total antioxidant capacity of organic Butternut squash samples during the industrial freezing process. The highest total antioxidant capacity values were obtained with the ABTS assay, followed by the CUPRAC, FRAP, and DPPH assays, respectively. The observed pattern could be attributed to the capability of the ABTS and CUPRAC assays to detect both hydrophilic and lipophilic antioxidants in foods. In contrast, the FRAP assay can exclusively detect hydrophilic antioxidants, while the DPPH assay is specific to lipophilic antioxidants [65]. The data obtained in this study suggest that applying multiple antioxidant capacity measurement assays with different mechanisms is necessary to reveal the complete capacity of an antioxidant. 

In summary, the goal of this study was to examine alterations in the bioaccessibility of carotenoids and polyphenols in organic Butternut squash throughout the industrial freezing process. Monitoring changes during the processing stages is crucial to elucidate at which stage the alteration in the final product originates. Thus, undesired changes occurring in the bioactive compounds of the product can be eliminated/reduced through optimization of the processing parameters. This approach would enable the production of final products with a high content of bioactive compounds on an industrial scale. To do so, we collected samples of organic Butternut squash from six different stages during the industrial freezing process. Assuming that washing and selection applied for a short time would not have a significant effect on the digestion of carotenoids and polyphenols, we did not take samples immediately before freezing. This may be a limiting factor to consider in future studies. Moreover, it is important to highlight that the procedures employed in this study yield semi-raw materials, which will undergo additional processing to reach the final product. Therefore, although this study offers valuable insights into the transformations during the production of industrially frozen squash, further research is necessary to comprehend the processing of these semi-raw materials into final baby food products. Another limitation of this study is the use of the adult digestion model. Considering the fact that some samples examined in this study (e.g., peel, pomace) may not be suitable for the consumption of infants as such, here we applied the standard adult digestion model. However, future studies should also consider the digestive conditions of infants and young children for the simulation of in vitro gastrointestinal digestion.

## 4. Conclusions

To the best of our knowledge, this study is the first to investigate the changes in the bioaccessibility of carotenoids and polyphenols in organic Butternut squash at different stages of the industrial freezing process. Overall, this study emphasized that industrial freezing could be an effective strategy for preserving the carotenoid bioaccessibility in organic Butternut squash, while also enhancing the levels of bioaccessible polyphenols. However, it is crucial to emphasize that the treatments employed in this study yield semi-raw materials to be processed further for final products. Therefore, future research should investigate the continual changes in the carotenoids and polyphenols of squash as they advance towards the production of the final baby food product. Moreover, this research focused exclusively on the impact of the freezing process, while it is also important to examine the changes in squash carotenoids and polyphenols during frozen storage. In addition, we also demonstrated that pomace and seed, which are discarded as waste, have significant potential to be utilized as a food source rich in bioactive compounds. However, additional research on toxicological data and the effect of the food matrix is necessary before these materials can be utilized as food ingredients. Finally, although often ignored, the consumer acceptance of food products containing waste materials also needs to be investigated.

## Figures and Tables

**Table 1:** Identification of the polyphenols detected in organic Butternut squash (*Cucurbita moschata*) samples by UPLC–ESI–MS/MS.

Retention Time	Ionization Mode	Mass (*m*/*z*)	MS^2^ Main Fragment (*m*/*z*)	Other Fragmental Ions (*m*/*z*)	Tentative Identification	Reference(s)
Flavonoids						
1.85	ESI+	306.9	139.0	289.0	Epigallocatechin	[52,53]
2.44	ESI-	289.1	245.1	205.1	Epicatechin	[49,50,51,52,53]
3.37	ESI-	447.1	285.0	256.0	Luteolin 7-*O*-glucoside (Cynaroside)	[48]
3.38	ESI-	463.1	300.1	301.1	Quercetin 3-*O*-galactoside (Hyperoside)	[48]
3.43	ESI-	477.1	301.0	151.1	Quercetin 3-*O*-glucuronide (Quercituron)	[51]
3.81	ESI-	578.9	271.1	459.2	Naringin	[48,49]
Phenolic acids						
1.74	ESI-	168.9	125.1	79.0	Gallic acid	[49,51,52,53]
2.01	ESI-	353.1	191.1	110.9	Chlorogenic acid	[49,52,53,54]
2.74	ESI-	197.0	123.1	121.1	Syringic acid	[48,51,54]
3.79	ESI-	192.9	134.1	178.1	Ferulic acid	[48,51,52,53,54]

**Table 2:** Changes in the bioaccessibility of flavonoids in organic Butternut squash (*Cucurbita moschata*) at six different stages of industrial freezing process.

Sample	Undigested	Oral Digestion	Gastric Digestion	Intestinal Digestion	Bioaccessibility (%)
Epigallocatechin (mg/kg)					
Raw material	58.7 ± 2.7 ^BC,a^	14.4 ± 3.0 ^C,c^	33.3 ± 6.9 ^B,bc^	45.2 ± 9.3 ^A,ab^	77
Skin	74.2 ± 6.0 ^B,a^	21.2 ± 0.3 ^B,c^	35.0 ± 1.6 ^B,b^	43.4 ± 3.5 ^A,b^	58
Pomace and seed	169.5 ± 10.2 ^A,a^	29.7 ± 0.1 ^A,c^	69.6 ± 12.6 ^A,b^	47.8 ± 6.9 ^A,bc^	28
Internal fruit	53.5 ± 1.6 ^CD^	<LOQ	<LOQ	<LOQ	n/a
Cutting	40.2 ± 3.5 ^D^	<LOQ	<LOQ	<LOQ	n/a
IQF	37.3 ± 1.1 ^D^	<LOQ	<LOQ	<LOQ	n/a
Epicatechin (mg/kg)					
Raw material	178.4 ± 5.8 ^BC,b^	49.5 ± 5.0 ^A,c^	58.2 ± 10.8 ^AB,c^	284.1 ± 51.9 ^B,a^	159
Skin	181.7 ± 6.0 ^B,a^	43.5 ± 3.8 ^AB,b^	73.5 ± 11.9 ^A,b^	173.2 ± 24.6 ^BC,a^	95
Pomace and seed	269.7 ± 19.7 ^A,b^	32.9 ± 5.8 ^BC,c^	71.1 ± 12.9 ^A,c^	454.0 ± 87.1 ^A,a^	168
Internal fruit	148.2 ± 3.2 ^C,a^	23.6 ± 6.0 ^CD,c^	27.6 ± 1.5 ^C,c^	105.8 ± 20.3 ^C,b^	71
Cutting	62.6 ± 1.4 ^D,a^	13.3 ± 2.7 ^D,b^	33.2 ± 5.5 ^BC,b^	80.3 ± 16.9 ^C,a^	128
IQF	74.8 ± 4.1 ^D,b^	14.6 ± 3.3 ^D,c^	24.8 ± 4.1 ^C,c^	117.5 ± 21.5 ^C,a^	157
Luteolin 7-*O*-glucoside (mg/kg)					
Raw material	5.1 ± 0.4 ^B^	<LOQ	<LOQ	<LOQ	n/a
Skin	6.3 ± 0.3 ^A^	<LOQ	<LOQ	<LOQ	n/a
Pomace and seed	<LOQ	<LOQ	<LOQ	<LOQ	n/a
Internal fruit	5.0 ± 0.1 ^B^	<LOQ	<LOQ	<LOQ	n/a
Cutting	<LOQ	<LOQ	<LOQ	<LOQ	n/a
IQF	<LOQ	<LOQ	<LOQ	<LOQ	n/a
Quercetin 3-*O*-galactoside (mg/kg)					
Raw material	18.0 ± 1.2 ^A^	<LOQ	<LOQ	<LOQ	n/a
Skin	16.4 ± 2.8 ^A^	<LOQ	<LOQ	<LOQ	n/a
Pomace and seed	13.3 ± 0.1 ^A^	<LOQ	<LOQ	<LOQ	n/a
Internal fruit	13.2 ± 0.1 ^A^	<LOQ	<LOQ	<LOQ	n/a
Cutting	<LOQ	<LOQ	<LOQ	<LOQ	n/a
IQF	<LOQ	<LOQ	<LOQ	<LOQ	n/a
Quercetin 3-*O*-glucuronide (mg/kg)					
Raw material	15.5 ± 0.8 ^A,b^	4.7 ± 1.2 ^A,c^	12.6 ± 2.1 ^A,b^	24.1 ± 1.8 ^A,a^	155
Skin	16.9 ± 0.1 ^A,ab^	3.1 ± 0.03 ^AB,c^	11.5 ± 2.8 ^A,b^	22.8 ± 3.0 ^A,a^	135
Pomace and seed	12.0 ± 0.1 ^B,b^	2.5 ± 0.01 ^B,d^	9.1 ± 0.1 ^A,c^	21.0 ± 0.6 ^A,a^	175
Internal fruit	<LOQ	<LOQ	<LOQ	<LOQ	n/a
Cutting	<LOQ	<LOQ	<LOQ	<LOQ	n/a
IQF	<LOQ	<LOQ	<LOQ	<LOQ	n/a
Naringin (mg/kg)					
Raw material	1.4 ± 0.04 ^B^	<LOQ	<LOQ	<LOQ	n/a
Skin	<LOQ	<LOQ	<LOQ	<LOQ	n/a
Pomace and seed	1.2 ± 0.04 ^C^	<LOQ	<LOQ	<LOQ	n/a
Internal fruit	1.6 ± 0.04 ^A^	<LOQ	<LOQ	<LOQ	n/a
Cutting	1.4 ± 0.02 ^B^	<LOQ	<LOQ	<LOQ	n/a
IQF	1.1 ± 0.01 ^C^	<LOQ	<LOQ	<LOQ	n/a

The values presented in this table are the mean values ± standard deviation obtained from three separate batches (n = 3). Statistically significant variations are indicated by uppercase letters in the columns and lowercase letters in the rows (*p* < 0.05). LOQ: Limit of quantification; n/a: not applicable.

**Table 3:** Changes in the bioaccessibility of phenolic acids in organic Butternut squash (*Cucurbita moschata*) at six different stages of industrial freezing process.

Sample	Undigested	Oral Digestion	Gastric Digestion	Intestinal Digestion	Bioaccessibility (%)
Gallic acid (mg/kg)					
Raw material	30.5 ± 1.1 ^B,a^	6.4 ± 2.0 ^A,b^	5.6 ± 1.3 ^A,b^	<LOQ	n/a
Skin	5.2 ± 0.3 ^C,a^	0.9 ± 0.2 ^B,c^	2.4 ± 0.3 ^B,b^	<LOQ	n/a
Pomace and seed	107.1 ± 4.2 ^A,a^	3.3 ± 0.2 ^B,c^	5.5 ± 1.1 ^A,bc^	12.7 ± 2.8 ^b^	12
Internal fruit	3.2 ± 0.1 ^C,a^	0.9 ± 0.2 ^B,b^	<LOQ	<LOQ	n/a
Cutting	3.9 ± 0.1 ^C,a^	0.5 ± 0.04 ^B,b^	<LOQ	<LOQ	n/a
IQF	1.7 ± 0.03 ^C,a^	0.6 ± 0.2 ^B,b^	<LOQ	<LOQ	n/a
Chlorogenic acid (mg/kg)					
Raw material	5.2 ± 0.3 ^B^	<LOQ	<LOQ	<LOQ	n/a
Skin	4.0 ± 0.02 ^C^	<LOQ	<LOQ	<LOQ	n/a
Pomace and seed	6.0 ± 0.2 ^A,b^	1.7 ± 0.2 ^c^	5.1 ± 0.5 ^b^	8.1 ± 0.7 ^a^	135
Internal fruit	4.3 ± 0.02 ^C,a^	1.0 ± 0.1 ^b^	<LOQ	<LOQ	n/a
Cutting	5.5 ± 0.2 ^AB,a^	1.7 ± 0.1 ^b^	<LOQ	<LOQ	n/a
IQF	4.3 ± 0.02 ^C,a^	1.1 ± 0.1 ^b^	<LOQ	<LOQ	n/a
Syringic acid (mg/kg)					
Raw material	43.7 ± 2.1 ^BC,a^	11.6 ± 1.1 ^d^	17.8 ± 2.1 ^A,c^	37.0 ± 1.6 ^B,b^	85
Skin	48.9 ± 3.8 ^B,a^	10.3 ± 0.8 ^b^	11.3 ± 0.8 ^B,b^	44.3 ± 8.1 ^B,a^	91
Pomace and seed	63.0 ± 4.4 ^A,b^	8.0 ± 1.3 ^c^	15.8 ± 2.0 ^A,c^	132.4 ± 20.1 ^A,a^	210
Internal fruit	36.2 ± 0.7 ^C,a^	5.9 ± 1.4 ^b^	8.6 ± 0.3 ^B,b^	29.4 ± 5.2 ^B,a^	81
Cutting	18.4 ± 1.8 ^D,ab^	<LOQ	8.9 ± 0.6 ^B,b^	27.5 ± 5.4 ^B,a^	149
IQF	20.0 ± 0.9 ^D,b^	<LOQ	11.4 ± 0.3 ^B,b^	32.0 ± 5.9 ^B,a^	160
Ferulic acid (mg/kg)					
Raw material	5.3 ± 0.4 ^A^	<LOQ	<LOQ	<LOQ	n/a
Skin	3.8 ± 0.1 ^B^	<LOQ	<LOQ	<LOQ	n/a
Pomace and seed	4.1 ± 0.03 ^B^	<LOQ	<LOQ	<LOQ	n/a
Internal fruit	3.8 ± 0.1 ^B^	<LOQ	<LOQ	<LOQ	n/a
Cutting	4.1 ± 0.1 ^B^	<LOQ	<LOQ	<LOQ	n/a
IQF	4.3 ± 0.1 ^B^	<LOQ	<LOQ	<LOQ	n/a

The values presented in this table are the mean values ± standard deviation obtained from three separate batches (n = 3). Statistically significant variations are indicated by uppercase letters in the columns and lowercase letters in the rows (*p* < 0.05). LOQ: Limit of quantification; n/a: not applicable.

**Table 4:** Changes in the bioaccessibility of total phenolics, flavonoids and antioxidant capacity in organic Butternut squash (*Cucurbita moschata*) at six different stages of industrial freezing process.

Sample	Undigested	Oral Digestion	Gastric Digestion	Intestinal Digestion	Bioaccessibility (%)
Total phenolic content (mg GAE/100 g)					
Raw material	57.9 ± 2.6 ^AB,b^	24.5 ± 1.9 ^B,c^	92.0 ± 2.4 ^B,a^	98.3 ± 9.7 ^AB,a^	170
Skin	49.6 ± 6.9 ^BC,b^	24.0 ± 0.2 ^B,c^	63.3 ± 3.5 ^CD,b^	85.2 ± 11.3 ^AB,a^	172
Pomace and seed	63.0 ± 6.0 ^A,b^	30.4 ± 1.9 ^A,c^	114.6 ± 6.4 ^A,a^	116.3 ± 22.0 ^A,a^	185
Internal fruit	44.8 ± 0.8 ^C,b^	19.4 ± 1.9 ^C,c^	68.6 ± 5.3 ^C,a^	74.1 ± 13.7 ^B,a^	165
Cutting	30.5 ± 1.3 ^D,bc^	14.9 ± 0.6 ^D,c^	47.8 ± 1.1 ^E,ab^	61.9 ± 17.1 ^B,a^	203
IQF	29.1 ± 5.1 ^D,b^	17.0 ± 0.7 ^CD,c^	53.9 ± 7.2 ^DE,a^	60.7 ± 2.0 ^B,a^	209
Total flavonoid content (mg RE/100 g)					
Raw material	7.6 ± 1.8 ^BC,bc^	3.8 ± 0.7 ^C,c^	9.5 ± 1.0 ^CD,b^	45.9 ± 2.8 ^B,a^	604
Skin	10.6 ± 0.8 ^AB,b^	6.7 ± 0.6 ^B,c^	22.0 ± 1.6 ^B,a^	13.3 ± 1.5 ^C,b^	125
Pomace and seed	13.1 ± 1.8 ^A,b^	10.5 ± 1.3 ^A,b^	30.1 ± 1.7 ^A,b^	122.4 ± 15.2 ^A,a^	934
Internal fruit	7.1 ± 1.1 ^BC,b^	2.6 ± 0.4 ^C,c^	12.7 ± 0.8 ^C,a^	8.6 ± 1.2 ^C,b^	121
Cutting	6.9 ± 1.5 ^BC,a^	2.2 ± 0.7 ^C,b^	5.7 ± 1.0 ^D,a^	8.1 ± 1.2 ^C,a^	117
IQF	6.6 ± 1.1 ^C,a^	2.3 ± 0.8 ^C,b^	9.5 ± 2.6 ^CD,a^	8.1 ± 1.1 ^C,a^	123
Total antioxidant capacity (mg TE/100 g)					
ABTS assay					
Raw material	55.1 ± 0.2 ^B,a^	10.8 ± 0.2 ^B,c^	35.0 ± 1.7 ^B,b^	62.6 ± 12.8 ^B,a^	114
Skin	44.1 ± 1.9 ^C,a^	9.5 ± 0.1 ^CD,c^	31.0 ± 2.9 ^BC,b^	42.2 ± 2.9 ^BC,a^	96
Pomace and seed	77.2 ± 1.4 ^A,b^	24.9 ± 0.2 ^A,c^	76.4 ± 0.1 ^A,b^	140.4 ± 8.0 ^A,a^	182
Internal fruit	44.4 ± 1.1 ^C,a^	10.0 ± 0.3 ^C,c^	27.2 ± 3.5 ^BC,b^	38.1 ± 4.3 ^C,a^	86
Cutting	35.4 ± 1.4 ^D,a^	9.7 ± 0.4 ^C,c^	23.4 ± 1.3 ^C,b^	28.2 ± 7.2 ^C,ab^	80
IQF	37.3 ± 1.0 ^D,a^	8.9 ± 0.5 ^D,c^	25.9 ± 6.5 ^BC,ab^	25.3 ± 5.7 ^C,b^	68
CUPRAC assay					
Raw material	36.5 ± 3.2 ^B,b^	15.3 ± 0.8 ^B,c^	40.6 ± 5.3 ^B,b^	64.1 ± 8.4 ^B,a^	176
Skin	24.3 ± 2.5 ^CD,bc^	11.5 ± 2.4 ^B,c^	41.6 ± 6.1 ^B,ab^	53.9 ± 12.0 ^B,a^	222
Pomace and seed	56.2 ± 6.1 ^A,b^	27.0 ± 5.8 ^A,b^	63.5 ± 11.9 ^A,b^	166.4 ± 43.6 ^A,a^	296
Internal fruit	25.4 ± 3.4 ^C,bc^	16.3 ± 1.4 ^B,c^	27.2 ± 2.1 ^B,b^	46.6 ± 7.0 ^B,a^	183
Cutting	16.6 ± 0.1 ^CD,b^	9.9 ± 0.8 ^B,c^	27.6 ± 2.8 ^B,a^	32.6 ± 3.3 ^B,a^	196
IQF	15.2 ± 2.1 ^D,b^	9.9 ± 3.3 ^B,b^	26.7 ± 4.0 ^B,a^	23.9 ± 2.2 ^B,a^	157
FRAP assay					
Raw material	11.6 ± 0.4 ^B,b^	2.6 ± 0.1 ^B,d^	8.0 ± 0.3 ^B,c^	31.3 ± 0.2 ^B,a^	270
Skin	5.4 ± 0.03 ^C,b^	1.7 ± 0.03 ^C,c^	7.7 ± 0.6 ^B,a^	7.3 ± 1.0 ^C,a^	135
Pomace and seed	26.9 ± 1.5 ^A,b^	4.0 ± 0.2 ^A,c^	20.7 ± 1.5 ^A,b^	91.8 ± 10.4 ^A,a^	341
Internal fruit	4.1 ± 0.3 ^CD,b^	2.7 ± 0.1 ^B,c^	5.4 ± 0.3 ^C,a^	5.6 ± 0.2 ^C,a^	137
Cutting	3.1 ± 0.3 ^D,a^	1.2 ± 0.1 ^D,c^	3.7 ± 0.2 ^CD,a^	2.3 ± 0.5 ^C,b^	74
IQF	2.2 ± 0.2 ^D,a^	0.9 ± 0.1 ^D,c^	2.8 ± 0.1 ^D,a^	1.5 ± 0.4 ^C,b^	68
DPPH assay					
Raw material	7.9 ± 1.3 ^B,b^	2.5 ± 0.2 ^B,c^	6.2 ± 0.8 ^B,b^	11.9 ± 0.1 ^B,a^	151
Skin	1.4 ± 0.1 ^C,b^	1.8 ± 0.6 ^BC,b^	1.3 ± 0.2 ^C,b^	7.6 ± 1.6 ^BC,a^	543
Pomace and seed	17.2 ± 1.6 ^A,b^	6.8 ± 0.7 ^A,c^	17.9 ± 3.8 ^A,b^	29.5 ± 5.7 ^A,a^	172
Internal fruit	2.5 ± 0.4 ^C,c^	1.9 ± 0.1 ^BC,c^	4.2 ± 0.5 ^BC,b^	7.2 ± 0.8 ^BC,a^	288
Cutting	0.5 ± 0.1 ^C,b^	1.8 ± 0.5 ^BC,ab^	2.2 ± 0.03 ^BC,a^	3.1 ± 1.0 ^C,a^	620
IQF	2.3 ± 0.7 ^C,b^	1.1 ± 0.1 ^C,b^	2.8 ± 1.2 ^BC,b^	7.0 ± 0.6 ^BC,a^	304

The values presented in this table are the mean values ± standard deviation obtained from three separate batches (n = 3). Statistically significant variations are indicated by uppercase letters in the columns and lowercase letters in the rows (*p* < 0.05).

## Data Availability

Data is contained within the article and Supplementary Materials.

## Supplementary Materials

The following supporting information can be downloaded at: https://www.mdpi.com/article/10.3390/foods13020239/s1, Table S1: Standards used for the quantification of polyphenols with HPLC-PDA.
