# Supplementary material for: Bioaccessibility of Carotenoids and Polyphenols in Organic Butternut Squash (Cucurbita moschata): Impact of Industrial Freezing Process
Source: Foods. 2024 Jan 11;13(2):239. doi: 10.3390/foods13020239 (PMC10814222; doi:10.3390/foods13020239)
Supplement: Supplementary file 1 [file foods-13-00239-s001.zip › foods-2789349-supplementary.pdf]

Table S1. Standards used for the quantification of polyphenols with HPLC-PDA.

**Compound**	**Slope**	**Intercept**	R^2	**LOD (mg/L)**	**LOQ (mg/L)**
Flavonoids					
Epigallocatechin	2545	-6387	0.9974	0.6	1.9
Epicatechin	6823	-3226	0.9999	0.5	1.7
Luteolin 7-*O*-glucoside (Cynaroside)	13330	-10919	0.9999	0.6	2.0
Quercetin 3-*O*-galactoside (Hyperoside)	15604	-39281	0.9981	2.7	9.1
Quercetin 3-*O*-glucuronide (Quercituron)	22443	-50988	0.9984	0.8	2.6
Naringin	15463	-2019	0.9994	0.8	2.7
Phenolic acids					
Gallic acid	26694	12696	0.9996	0.4	1.4
Chlorogenic acid	23847	-18436	0.9999	0.8	2.8
Syringic acid	30921	-24654	0.9999	0.4	1.2
Ferulic acid	49055	-33126	0.9999	0.7	2.2

LOD: limit of detection, LOQ: limit of quantification.
